# Time-Course Effects of Acute Aflatoxin B1 Exposure on Hepatic Mitochondrial Lipids and Oxidative Stress in Rats

**DOI:** 10.3389/fphar.2019.00467

**Published:** 2019-05-07

**Authors:** Oluwakemi A. Rotimi, Solomon O. Rotimi, Jaclyn M. Goodrich, Isaacson B. Adelani, Emmanuel Agbonihale, Gbemisola Talabi

**Affiliations:** ^1^Department of Biochemistry and Molecular Biology Research Laboratory, Covenant University, Ota, Nigeria; ^2^Department of Environmental Health Sciences, University of Michigan, Ann Arbor, MI, United States

**Keywords:** aflatoxin B1, liver, mitochondria, lipid, cholesterol, thioredoxin reductase, oxidative stress

## Abstract

Aflatoxins are secondary metabolites of certain *Aspergillus* species, that contaminate staple foods, particularly in developing countries. Aflatoxin B1 (AFB1) is the most toxic and common of the major types of aflatoxins. AFB1 is hepatotoxic and has been implicated in increasing the risk of hepatocellular carcinoma (HCC). We have previously shown that subacute exposure to AFB1 for 7 days disrupts hepatic lipids; therefore, this study determined the time-course effects of acute aflatoxin exposure on hepatic mitochondrial lipids and oxidative stress. To achieve this, thirty male albino rats were randomly assigned to six groups. The groups received an oral dose of 1 mg/kg body weight AFB1 or vehicle only (controls) for one, four, or seven days, respectively. Twenty-four hours after the last dose, the animals were sacrificed and liver excised. Mitochondria and cytosolic fractions were obtained from the liver after which lipids (cholesterol, triacylglycerols) were determined in the mitochondria while biomarkers of oxidative stress (glutathione, glutathione transferase (GST), glutathione peroxidase (GPx), glutathione reductase, nitric oxide (NO), malonaldehyde (MDA), thioredoxin reductase (TR), and superoxide dismutase (SOD) were determined spectrophotometrically in the mitochondria and cytosolic fractions. The expression of genes (*Nrf2*, *Acc*, *Nqo1*, and *HmgCoa*) were determined using quantitative RT-PCR. Results showed that AFB1 significantly increased mitochondrial cholesterol at day seven (treatment vs. control, *p* = 0.016). It also increased the concentrations of NO and MDA at day one and day seven while the activity of GPx and concentration of GSH were increased at day seven (*p* = 0.030) and day one (*p* = 0.025) alone, respectively, compared to control. The activities of cytosolic GR (*p* = 0.014), TR (*p* = 0.046) and GST (*p* = 0.044) were increased at day seven. AFB1 significantly increased the expression of *Nrf2* (*p* = 0.029) and decreased the expression of *Acc* (*p* = 0.005) at day one. This study revealed that AFB1 disrupts hepatic mitochondrial lipids and antioxidant capacity. These changes were dependent on the timing of exposure and did not follow a linear time-course trend. These alterations could be part of the hepatic mitochondria response mechanism to acute AFB1 toxicity.

## Introduction

Aflatoxins are compounds that are produced as secondary metabolites of fungi belonging to the *Aspergillus* species particularly *Aspergillus flavus*, *parasiticus*, and *nominus* (Mutegi et al., 2018). These fungi thrive well in the Tropical climate where they grow on foodstuffs from farm to table (Gong and Watson, 2016; Mutegi et al., 2018). Hence, aflatoxins contaminate food and feed stuff especially cereals and nuts in sub-Saharan Africa and southeast Asia (Wild and Gong, 2009; Gong and Watson, 2016; Opoku et al., 2018; Rushing and Selim, 2018). There are about 18 types of aflatoxins; however, the major ones are AFB1, AFB2, AFG1, AFG2, AFM1, and AFM2 (Luo et al., 2018). Of these, AFB1 is the most toxic and prevalent and has been named a class 1 carcinogen by the International Agency for Research on Cancer (IARC) (Ostry et al., 2017). AFB1 has been shown to affect several organs and tissues; however, the liver is its target organ, where it has been shown to cause hepatocellular carcinoma (HCC) after chronic exposure (Wogan et al., 2012; Rushing and Selim, 2018). It is also mutagenic, teratogenic, immunotoxic (Bondy and Pestka, 2000; Rushing and Selim, 2018), impairs growth in children (Khlangwiset et al., 2011; Watson et al., 2018) and affects reproductive health (Eze et al., 2018).

AFB1 is usually converted by the microsomal CYP_450_ family of enzymes to a more toxic epoxide, AFB1-8,9-epoxide (AFBO) (Deng et al., 2018). This highly reactive epoxide is responsible for the majority of the tissue toxicity of AFB1 because it binds to macromolecules such as DNA and proteins (Rushing and Selim, 2018). To be fully detoxified, the epoxide crosses the mitochondria membrane where it goes through phase II metabolism by the detoxification enzymes such as glutathione transferases (GSTs) (Deng et al., 2018). Apart from its detoxification functions, the mitochondrion is also involved in other biological processes such as the oxidation process by the electron transport chain and production of reactive oxygen species (Bhat et al., 2015; Wang et al., 2017). Hence, the critical roles that mitochondria play in the metabolism and detoxification of aflatoxin make it important in the pathogenesis of aflatoxicosis and therefore requires detailed investigation. Previous studies have shown that AFB1 impairs mitochondria function such as mitochondrial permeability (Liu and Wang, 2016; Wang et al., 2017). The permeability of the membrane is due in part to membrane transporters and its lipid composition (Van Meer et al., 2008). We and others have previously shown that AFB1 disrupts lipid metabolism and induces oxidative stress (Lu et al., 2013; El-Nekeety et al., 2014; Rotimi et al., 2016, 2017, 2018; Zhang et al., 2016). The balance of homeostasis within these two biochemical processes is important to the optimal function of mitochondria.

Although acute aflatoxicosis occurs only occasionally when compared with chronic exposure, such acute exposures, which often lead to death, have been reported in Kenya and Tanzania (Ngindu et al., 1982; Azziz-Baumgartner et al., 2005; Lewis et al., 2005; Kamala et al., 2018). Hence, it is important to understand the time-course events associated with acute exposure to AFB1. Due to the essential role of mitochondria in the detoxification of AFB1 and pathogenesis of aflatoxicosis, we hypothesize that mitochondria oxidative stress and dyslipidemia will be associated with it in a time-dependent manner. The aim of this study, therefore, was to investigate the time course effects of subacute AFB1 hepatotoxicity in albino Wistar rats. We achieve this by studying the changes in liver function, mitochondrial lipids and oxidative stress as well as the expression of genes associated with lipid metabolism and oxidative stress.

## Materials and Methods

### Chemicals

AFB1 and other chemicals including mannitol, sucrose, HEPES, succinate, ethylenediaminetetraacetic acid (EDTA) used in this study were products of Sigma–Aldrich (St. Louis, MO, United States). The RNA extraction kit was a product of Aidlab^®^ Biotechnologies Co., Ltd., (Beijing, China).

### Animals

Thirty male inbred albino Wistar rats weighing between 100–120 g were used for this study. They were housed at room temperature and allowed access to food and water *ad libitum*. The animals were acclimatized to the experimental condition for 2 weeks before the commencement of exposure. The experiment was conducted, and the animals cared for according to the institutional animal care and use committee guidebook and as approved by the Covenant University Ethical committee.

### Experimental Design

The animals were randomly divided into six groups of five animals each, and they received an oral gavage dose of AFB1 (1 mg/kg body weight) in olive oil or olive oil only (controls) once daily for one, four, or seven days as shown below;

AFB1 day 1: 1 mg/kg AFB1 for one day.

Control day 1: Olive oil for one day.

AFB1 day 4: 1 mg/kg AFB1 for four days.

Control day 4: Olive oil for four days.

AFB1 day 7: 1 mg/kg AFB1 for seven days.

Control day 7: Olive oil for seven days.

The animals were sacrificed 24 h after the last dose and blood was collected by cardiac puncture into heparinized tubes. A portion of the liver was kept in RNAlater^®^ (Qiagen) for RNA extraction while the remaining part was immediately transferred into the mitochondria isolation buffer. The blood was centrifuged at 3000 rpm for 15 min to obtain the plasma.

### Preparation of Liver Mitochondria

Liver mitochondria were isolated as described by Gogvadze et al. (2004). The liver was removed immediately after sacrifice and rinsed in freshly prepared ice-cold (4°C) homogenizing buffer (210 mM mannitol, 70 mM sucrose, 5 mM HEPES, and 1 mM EDTA pH 7.5) to remove blood. A 10% homogenate was then prepared after which the homogenate was centrifuged (600 × *g*, 8 min, 4°C) to remove nuclei and unbroken cells. The resulting supernatant was centrifuged (5500 × *g*, 15 min, 4°C) to form the mitochondria pellet while the supernatant represented the cytosolic fraction. The pellet was suspended in about 0.3 ml of the buffer and then diluted with the homogenizing buffer (excluding 1 mM EDTA) after which it was centrifuged again (5500 × *g*, 15 min, 4°C). The final mitochondria pellet was suspended in about 1 ml of the same buffer and protein concentration was determined according to the method of Lowry et al. (1951). The resulting mitochondrial and cytosolic fractions were used for further analysis.

### Biochemical Analysis

#### Biomarkers of Liver Function

Alkaline phosphatase (ALP), alanine transaminase (AST), and bilirubin were determined spectrophotometrically using Randox kits according to manufacturer’s instructions.

#### Mitochondrial Cholesterol and Triacylglycerol

Lipid was extracted from the liver mitochondria according to the method of Folch et al. (1957). The extract obtained was used for determining triacylglycerol and cholesterol spectrophotometrically, using commercially available kits, as described by Rotimi et al. (2012).

#### Mitochondrial and Cytosolic Oxidant/Antioxidant Status

Malondialdehyde (MDA) concentration was determined using the method of Buege and Aust (1978) by measuring the concentration of thiobarbituric acid reactive substances (TBARS). Nitric oxide (NO) concentration was determined using a method described by Yucel et al. (2012). Glutathione (GSH) concentration was determined using the method described by Ellman (1959), while glutathione peroxidase (GPx) activity was determined using the method of Rotruck et al. (1973). Glutathione S-transferase (GST) activity was determined according to the method of Habig et al. (1974) while superoxide dismutase (SOD) activity was determined using the pyrogallol autoxidation method by Marklund and Marklund (1974). Glutathione reductase (GR) was assayed using oxidized glutathione (GSSG) in the method described by Mavis and Stellwagen (1968) while thioredoxin reductase (TR) was determined as described by Holmgren and Bjornstedt (1995).

#### Mitochondrial ATPase, NADH-Cytochrome C Reductase and Succinate Dehydrogenase

Mitochondrial total ATPase was determined using the Taussky and Shorr, 1953 method. Mitochondrial sample equivalent to 50 μg protein was added to a reaction mixture of 2 M NaCl solution and 24 mM Tris–HCl Buffer containing 0.68 mM EDTA and 6.0 mM MgCl_2_. The mixture was centrifuged and the supernatant obtained was added to Taussky-Shorr reagent. The mixture which was incubated at 25°C for 5 min after which absorbance was taken at 660 nm.

Succinate dehydrogenase assay was carried out using the method described by Reisch and Elpeleg (2007). Mitochondrial sample equivalent to 10 μg protein was preincubated in 0.1 M phosphate buffer (pH 7.4) with 0.2 M succinate for 20 min at 25°C. After incubation, 0.2 M potassium cyanide, freshly prepared 5 mM 2,6-Dichlorophenolindophenol and 65 mM phenzine methosulfate were added and absorbance read at 660 nm.

NADH – Cytochrome C reductase (complex I-III) was determined as described by Navarro et al. (2004). Mitochondria sample equivalent to 50 μg protein was added to 0.1 M phosphate buffer (pH 7.4) containing 0.2 mM NADH and 1 mM KCN. The reaction was then initiated by the addition of 0.1 mM cytochrome C and absorbance was measured every minute for 3 min at 550 nm.

#### Extraction of RNA and Real-Time Quantitative Polymerase Reaction(RT-qPCR)

RNA was extracted from the liver as previously described by Rotimi et al. (2017) after which the concentration and purity were checked using a NanoDrop spectrophotometer. 0.5 μg of RNA was reverse transcribed for complementary DNA (cDNA) synthesis using Bio Rad iScript cDNA. The cDNA was diluted 1:10 after which the qPCR was performed on the Bio Rad CFX96 Real-time PCR detection system using iQ SYBR Green Supermix kit and gene-specific primers for *Nrf2*, *Acc*, *Hmgcoa*, and *Nqo1* genes (Table 1). The samples were analyzed in triplicates and two housekeeping genes (β-*actin* and *Gapdh*) and an internal control were used. The relative expression of the genes was calculated using the 2^-ΔΔ^*^Ct^* method (Livak and Schmittgen, 2001).

**Table 1:** Sequence of gene specific primers for RT-qPCR.

Gene	Sequence (5′–3′)	References
*Nrf2*	Forward: TAGATGACCATGAGTCGCTT	Sun et al., 2017
	Reverse: CTGTAACTCGGGAATGGAAA	
*Hmgcr*	Forward: CAACCTTCTACCTCAGCAAGC	Su et al., 2016
	Reverse: ACAGTGCCACACACAATTCG	
*Acc*	Forward: TGAGGAGGACCGCATTTATC	Harasiuk et al., 2016
	Reverse: CCACAGCAATGGCAGGACTA	
*Nqo1*	Forward: GAAGCTTCCTTCGTGACCAG	Sun et al., 2017
	Reverse: GGGGGTTAAAGTTCATAGCA	
*Gapdh*	Forward: GGCAAGTTCAATGGCACAGT	Suzuki et al., 2016
	Reverse: TGGTGAAGACGCCAGTAGACTC	
β-*Actin*	Forward: AGCGTGGCTACAGCTTCACC	Julian et al., 2014
	Reverse: AAGTCTAGGGCAACATAGCACAGC	


### Statistical Analysis

Results were analyzed using SPSS. Two-tailed *T*-test was used to determine the difference between the control and treatment group at each time point. ANOVA was used to determine the difference between all the groups with post hoc pairwise comparisons by Duncan’s multiple range test.

## Results

**FIGURE 1:**
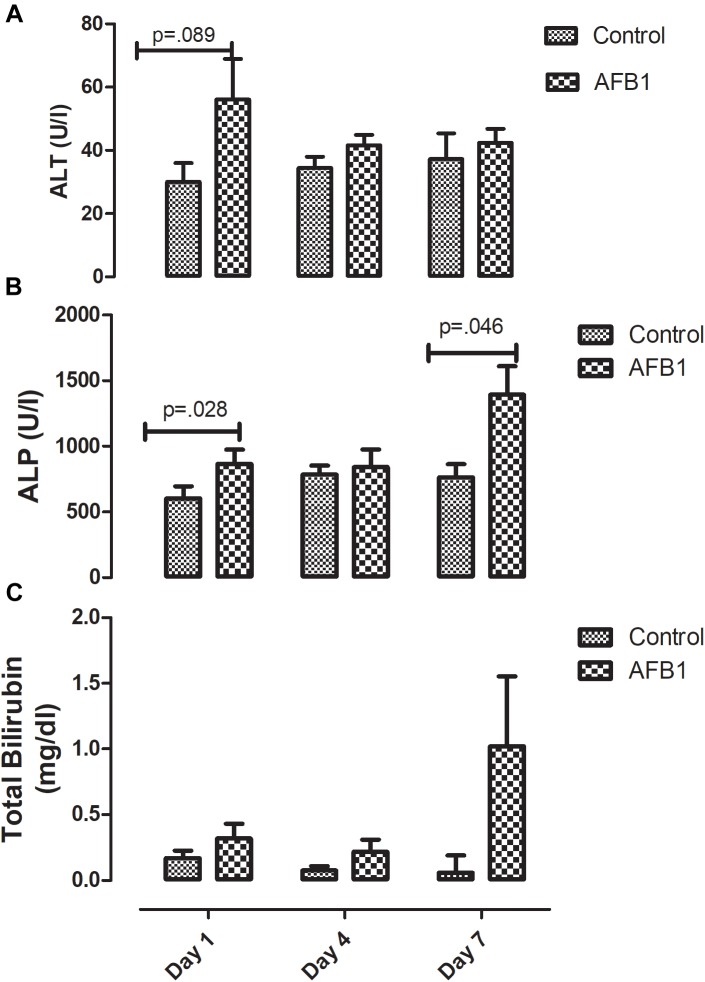
Time course effects of sub-acute AFB1 exposure on **(A)** ALT, **(B)** ALP, and **(C)** total bilirubin. *P*-values <0.1 are shown for comparisons between control and AFB1 at a given time point, and *p* < 0.05 is considered significant. Bars represent mean ± SEM (*n* = 5).

Time course effects of subacute AFB1 exposure significantly increased plasma ALP (Figure 1B) at day 1 (control vs. treatment) (*p* = 0.028) and day 7 (*p* = 0.046) while there was no significant difference in ALT (Figure 1A) between the control and treatment groups at all time points. Although there was no statistically significant difference in plasma bilirubin (control vs. treatment) at any time point, bilirubin concentration resulting from AFB1 treatment for 7 days was significantly (*p* < 0.05) higher than that of day 1 and/or 4 days (Figure 1C).

**FIGURE 2:**
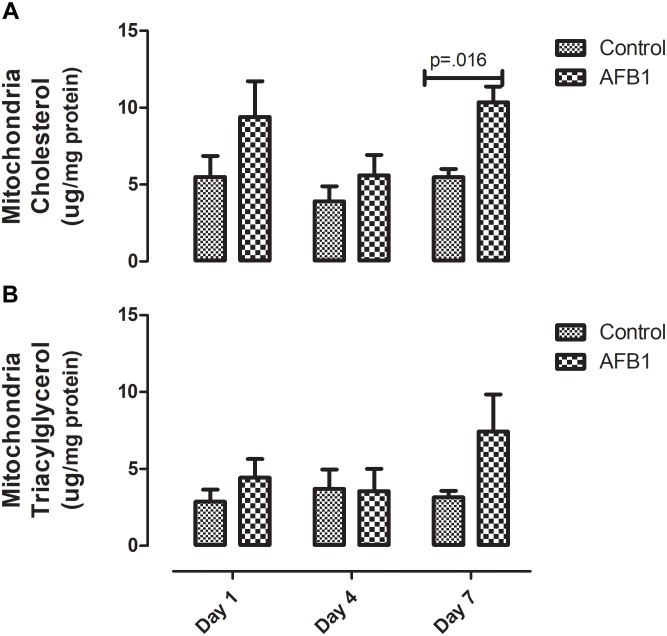
Time course effects of sub-acute AFB1 exposure on mitochondrial **(A)** cholesterol and **(B)** triacylglycerol. Significant *p*-values (*p* < 0.05) for control vs. AFB1 at a given time point are shown. Bars represent mean ± SEM (*n* = 5).

**FIGURE 3:**
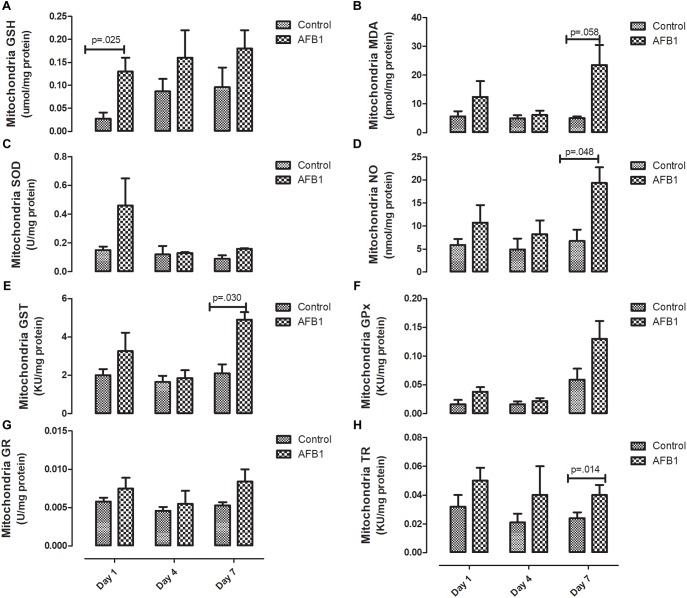
Time course effects of sub-acute AFB1 exposure on mitochondrial **(A)** GSH, **(B)** MDA, **(C)** SOD, **(D)** NO, **(E)** GST, **(F)** GPx, **(G)** GR, and **(H)** TR. *P*-values <0.1 are shown for comparisons between control and AFB1 at a given time point, and *p* < 0.05 is considered significant. Bars represent mean ± SEM (*n* = 5).

Sub-acute AFB1 exposure disrupts mitochondrial lipids and oxidative stress. AFB1 (1.0 mg/kg) significantly increased mitochondrial cholesterol (*p* = 0.016) after exposure for 7 days while there was no significant difference in mitochondrial triacylglycerols (Figure 2). The time course effects of subacute AFB1 exposure on mitochondrial oxidant/antioxidant status are depicted in Figure 3. AFB1 exposure significantly increased GSH (*p* = 0.025) at day 1 while NO (*p* = 0.048) and MDA (*p* = 0.058) were increased at day 7. GST (*p* = 0.030) and TR (0.014) were also significantly increased as a result of AFB1 exposure after 7 days of exposure. NO, MDA, GST, and GPx of the treatment group at day 7 was significantly (*p* < 0.05) higher than that of day 4. There was no significant difference in mitochondrial SOD on all the days.

**FIGURE 4:**
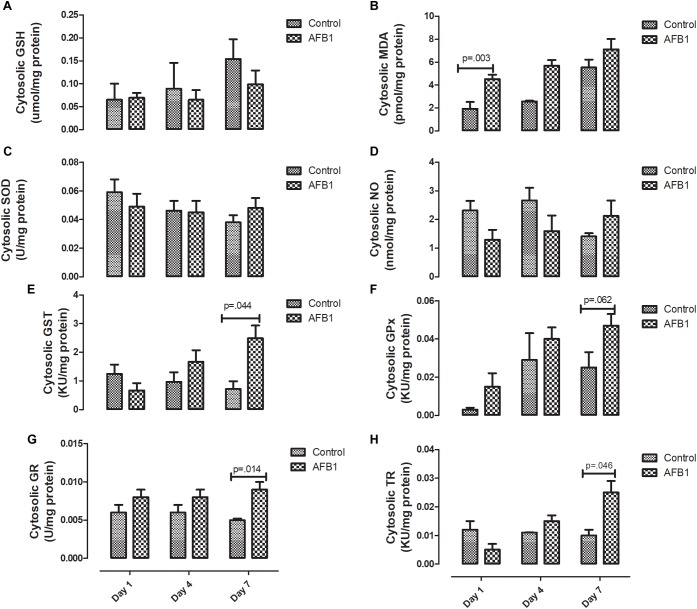
Time course effects of sub-acute AFB1 exposure on cytosolic **(A)** GSH, **(B)** MDA, **(C)** SOD, **(D)** NO, **(E)** GST, **(F)** GPx, **(G)** GR, and **(H)** TR. *P*-values <0.1 are shown for comparisons between control and AFB1 at a given time point, and *p* < 0.05 is considered significant. Bars represent mean ± SEM (*n* = 5).

The time course effects of subacute AFB1 exposure on the oxidant/antioxidant status of the cytosolic fractions are depicted in Figure 4. AFB1 significantly increased MDA at only day 1 (*p* = 0.003) while GST (*p* = 0.044), GR (0.014), and TR (0.046) were significantly increased at day 7. In the cytosolic fractions, AFB1 exposure at day 7 significantly (*p* < 0.05) increased MDA, GST, TR, and GPx when compared with AFB1 exposure at day 1. There was no significant difference in cytosolic SOD on all the days.

**FIGURE 5:**
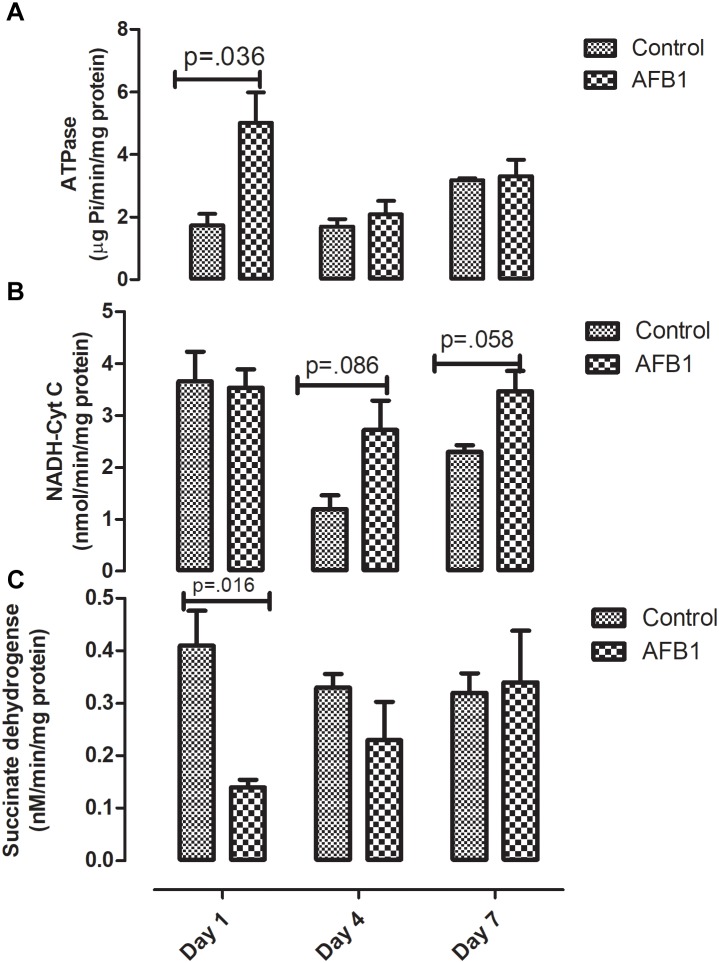
Time course effects of sub-acute AFB1 exposure on mitochondrial **(A)** ATPase, **(B)** NADH-Cyt C, and **(C)** Succinate dehydrogenase. *P*-values <0.1 are shown for comparisons between control and AFB1 at a given time point, and *p* < 0.05 is considered significant. Bars represent mean ± SEM (*n* = 5).

Time course subacute AFB1 exposure on mitochondrial enzymes are shown in Figure 5. AFB1 significantly increased ATPase (*p* = 0.036) while it decreased succinate dehydrogenase at day 1 (*p* = 0.016). AFB1 also increased NADH-Cyt C (*p* = 0.058) at day 7 only.

**FIGURE 6:**
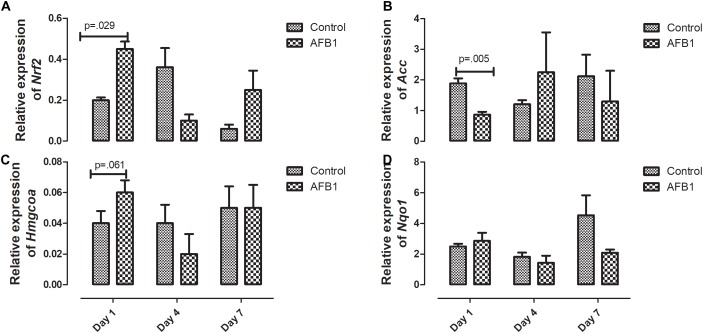
Time course effects of sub-acute AFB1 exposure on relative expression of **(A)** *Nrf2*, **(B)** *Acc*, **(C)** *Hmgcoa*, and **(D)** *Nqo1* in liver. *P*-values <0.1 are shown for comparisons between control and AFB1 at a given time point, and *p* < 0.05 is considered significant. Bars represent mean ± SEM (*n* = 3).

The time course effects of AFB1 on the relative expression of genes associated with oxidative stress are shown in Figure 6. AFB1 increased *Nrf2* (*p* = 0.029) and *Hmgcoa* (*p* = 0.061) at day 1 while *Acc* (0.005) was significantly decreased at day 1.

## Discussion

Time-course effects of subacute AFB1 exposure for 1, 4, and 7 days increased biomarkers of liver function, mitochondrial cholesterol, mitochondrial oxidant status (MDA and NO) as well as increased the enzymic and non-enzymic antioxidant status. These changes were associated with upregulation of *Nrf2* and downregulation of *Acc* at day 1. These changes were dependent on the timing of exposure but did not follow a linear time-course trend. Most of the changes observed in this study were at day 1 (initial exposure) or at day 7 (last exposure). The changes at day 1 may be an initial response to toxic AFB1. This initial response was characterized by increases in liver ALP, mitochondrial GSH concentrations, ATPase activity, cytosolic MDA, liver *Nrf2* expression, and decreases in mitochondrial succinate dehydrogenase activity and liver *Acc* expression. The changes collectively, may represent the initial protective response of the mitochondria against the aflatoxin toxicity. At the end of the time course, changes between exposed and controls included increases in ALP and bilirubin, mitochondrial MDA, NO, and GST activity, and cytosolic GST, GR, and TR activity. These outcomes may reflect an overwhelmed response system with increased toxicity manifesting in the liver.

In this study, AFB1 significantly increased the activity of ALP at day 1 and day 7 when compared with controls while the concentration of bilirubin in AFB1 exposed group at day 7 was significantly higher than those of day 1 and day 4. The concentrations of ALT, ALP, and bilirubin are usually increased in the plasma in response to hepatic injury which allows them to leak out into the plasma. Although plasma ALT activity is considered a gold standard for measuring liver injury, ALP, GGT, and total bilirubin are biomarkers used in addition to ALT especially in relation to conventional biliary action. Studies have shown that during biliary cirrhosis, there could be increases in ALP and GGT without corresponding increase in ALT (Ramaiah, 2007; Ozer et al., 2008). Although we did not assay for GGT in this study, the increase in ALP and bilirubin observed in this study suggests that liver injury may be associated with the hepatobiliary action and tends towards cholestatic induction. During the progression of cholestasis, increases in cholestatic induction enzymes such as ALP usually precedes an increase in bilirubin (Ramaiah, 2007). Several studies have reported increases in these biomarkers following AFB1 exposure (El-Nekeety et al., 2014; Rotimi et al., 2016; Eftekhari et al., 2018). Redzwan et al. (2014) found a positive correlation between AFB1-lysine adducts and total bilirubin in Malaysian adults. While ALP is localized within the cell membrane of tissues, ALT could be found in the cytoplasm and mitochondria, and changes in membrane permeability due to lipid accumulation could allow these enzymes to leak out into the cytosol.

AFB1 significantly increased mitochondrial cholesterol at day 7. Of all cellular compartments, the mitochondrion is considered to have low cholesterol concentration. It contains about 0.5–3% of cholesterol found in other organelles (Ikonen, 2008; Van Meer et al., 2008). Cholesterol is an essential component of the mitochondrial membrane, and increased accumulation of cholesterol in the mitochondria decreases its membrane’s fluidity and consequently its permeability (Ikonen, 2008; Van Meer et al., 2008). The increase in mitochondrial cholesterol observed in this study may be one of the mechanisms by which AFB1 affects the mitochondria and induces toxicity. Increase in mitochondrial cholesterol has been suggested to aggravate cholestasis (Nuño-Lámbarri et al., 2016). Apart from cholestasis, recent evidence showed that elevated mitochondria cholesterol might be involved in other diseases associated with the liver such as steatohepatitis and progression of HCC (García-Ruiz et al., 2016). Therefore, elevated mitochondria cholesterol may be one of the events associated with AFB1 induced HCC. Since we report evidence for this after only 7 days of exposure, this needs to be further studied in a chronic exposure experiment beyond 7 days. It is worthy of note that the increase in mitochondrial cholesterol was not associated with increased expression of *Hmgcr*. Although there was no significant increase in expression of *Hmgcr* gene at day 7, a nearly significant (*p* = 0.061) increase was observed at day 1. Vohra et al. (1985) found that acute AFB1 exposure increased mitochondrial cholesterol in rats after a single dose with higher concentration (5 mg/kg) given intraperitoneally.

Elevated mitochondria cholesterol negatively affects several membrane carriers. An example is the GSH transport system responsible for the transport of GSH from the cytosol- where it is synthesized- to the mitochondria through an ATP-dependent process (Fernandez-Checa and Kaplowitz, 2005). In this study, mitochondrial GSH (mGSH) was significantly increased at day 1 while there was no significant difference at day 7. The increase in GSH at day 1 also corresponds with the increase in ATPase observed at day 1. Although the transport of GSH is controlled by mitochondrial cholesterol concentration through an ATP-dependent process, it is worthy of note that the changes in mitochondrial cholesterol, GSH and ATPase did not occur at the same time. It is possible that these changes precede one another, while significant changes in GSH and ATPase were observed at day 1, significant increases in cholesterol were observed at day 7.

GSH together with mitochondrial antioxidant enzymes are responsible for maintaining the oxidant/antioxidant status of the mitochondria considering its role in electron transport chain and the generation of free radicals (reactive oxygen/nitrogen species) (Bhat et al., 2015). In this study AFB1 induced mitochondrial oxidative/nitrosative stress as shown by the increase in mitochondrial MDA and NO at day 7. Surprisingly, the increase in MDA and NO were associated with an increase in mitochondria GST and TR. It is expected that increase in MDA and NO will be associated with a decrease in antioxidant enzymes; however, it seems that the increase in GST and TR observed may be an attempt to counter the increase in oxidative/nitrosative species in this acute exposure. During aflatoxin metabolism, GST catalyzes the conjugation of AFBO with GSH for onward detoxification. The observed increase in GST may be a protective measure in response to subacute AFB1 exposure.

In the same vein, although increase in thioredoxin reductase (TR) seems to play a role in protecting against oxidative stress, other studies show that thioredoxin in reduced form may support the growth of cancer cells by complexing with apoptosis signaling kinase 1 (ASK1), which then prevents apoptosis (Arnér and Holmgren, 2000). Rieswijk et al. (2016) found that repetitive AFB1 (0.3 μM) exposure for 5 days led to persistent hypomethylation and upregulation of TR-1 –which has been shown to be associated with AFB1 and related to the development of HCC- in primary human hepatocytes (Caiment et al., 2014). Our study showed that AFB1 significantly increased the activity of mitochondrial and cytosolic TR at day 7. Considering the similar increase observed in other antioxidants in this study, it seems that the increase in TR activity plays a role in preventing against AFB1-induced oxidative stress.

On the other hand, this increased activity may be as a result of AFB1-induced hypomethylation and upregulation of TR (Rieswijk et al., 2016) and adequate availability of selenium (a cofactor of TR) whose deficiency has been shown to protect against aflatoxicosis in rats (McLeod et al., 1997). To the best of our knowledge, this study is the first to report the activity of TR in response to subacute AFB1 exposure in rats. Zhang et al. (2016), using RNA-Seq, studied the hepatic transcriptome of ducklings in response to AFB1 after 2 weeks of exposure. They found that *TR*, GSTs, and *Nqo1* were upregulated while *Acc* was down-regulated. This is similar to the results observed in this study, our results showed that AFB1 downregulated *Acc* at day 1 only. *Acc* is associated with the promotion of fatty acid synthesis, and we have previously shown that subacute exposure to AFB1 for seven days increased plasma free fatty acid (FFA) due to downregulation of CPT1 necessary for transporting fatty acids into the mitochondria for ß oxidation (Rotimi et al., 2017).

*Nrf2* is a transcription factor usually activated in response to reactive oxygen species. Apart from its role in antioxidant defense, it has also been shown to play a role in hepatic lipid metabolism (Shimpi et al., 2017). In this study, we found that AFB1 upregulated the expression of *Nrf2* at day 1 alone. This increase was in response to increased oxidant status and an attempt to maintain the oxidant/antioxidant status balance. *Nrf2* does this by upregulating other antioxidant enzymes particularly the phase II detoxification enzymes. Although we did not determine the expression of these enzymes, their activities were increased. The increase in *Nrf2* was associated with increased GST both in the mitochondrial and cytosolic compartments. AFB1 (0.5–5 μmol/L) upregulated *Nrf2* in broiler hepatocytes and cardiomyocytes after exposure for 24–72 h (Liu and Wang, 2016; Wang et al., 2017). Recently, Muhammad et al. (2018) found that exposure of broilers to AFB1 (5 mg/kg feed) for 28 days significantly downregulated *Nrf2*. Their result differs from ours, and this could be a result of exposure (subacute vs. subchronic) and species (rat vs. broiler) differences.

In this study AFB1 significantly decreased hepatic succinate dehydrogenase (SDH) at day 1. Our results are similar to other studies such as that of Rastogi et al. (2001) where they observed a 16% decrease in SDH after a single dose of AFB1 (2 mg/kg) in rats. A similar trend was also observed in birds (Quezada et al., 2000). Acute AFB1 exposure of rats to a single dose of AFB1 (7 mg/kg bw) significantly decreased SDH after 12 h of exposure after which the activity of SDH returned to normal (Sajan et al., 1995). Vohra et al. (1985) also observed a significant decrease in the activity of liver SDH 6 to 24 h after a single dose of AFB1 (5 mg/kg) in rats. They observed that the effect of AFB1 on SDH was time-dependent and maximum decrease was observed 24 h after treatment.

Strengths of this study include, time course and the biomarkers used to assess oxidative damage particularly TR activity, which has been reported to play a dual role in protecting against oxidative damage but also in driving cellular carcinogenesis. This study was limited by the sample size (5 in each group) which may have limited our statistical power to detect all true significant differences between groups. Although the dosage used is higher than that reported in human exposure; our previous study established this dosage to be adequate for eliciting toxic response in rats, without causing death. In addition, we analyzed expression of four genes implicated in response to AFB1 out of many possible. Future research should assess the effects of acute AFB1 over several days or weeks on animals of both sexes and multiple ages (adults and young) to determine critical windows in the time course of toxicity that mimic populations exposed to highly contaminated foods.

## Conclusion

In conclusion, subacute exposure to AFB1 showed different sets of responses at day 1 and after 7 days of exposure. We showed that AFB1 significantly increased biomarkers of liver functions and oxidative stress which was associated with increased mitochondrial lipids. Interestingly, we also observed an increase in mitochondrial antioxidant enzymes and particularly, we showed for the first time that subacute AFB1 exposure increased thioredoxin reductase activity in both cytosolic and mitochondrial compartments.

## Ethics Statement

This study experiment was conducted, and the animals cared for in accordance with the institutional animal care and use committee guidebook. The protocol was approved by the Department of Biological Sciences, Covenant University Ethical committee.

## Author Contributions

OR and SR conceived, designed, and supervised the experiments. OR and JG designed and carried out the gene expression assays. IA, EA, and GT carried out the mitochondrial assays. OR drafted the manuscript. All authors contributed to revision and approved the manuscript.

## Conflict of Interest Statement

The authors declare that the research was conducted in the absence of any commercial or financial relationships that could be construed as a potential conflict of interest.
